# 50 Years of metabolic control analysis

**DOI:** 10.1098/rsfs.2023.0080

**Published:** 2024-02-09

**Authors:** Herbert M. Sauro

**Affiliations:** Department of Bioengineering, University of Washington, PO Box 355061, Seattle, WA 98195-5061, USA

It has been 50 years since the publication of the seminal works by Savageau [1], Kacser & Burns [2,3] and Heinrich & Rapoport [4], on the control of biochemical networks. What is remarkable about these three publications (especially the two papers by Kacser *et al*. and Rapoport *et al*.) is that they independently developed almost the same approach and came to the same conclusions. This approach is today called metabolic control analysis (MCA), and is a body of work that uses mathematics to reason about the properties of biochemical pathways in a deductive manner. Although originally focused on metabolic pathways, it was realized early on that it could be equally applied to genetic and signalling networks [5,6]. In fact anything that one could associate a stoichiometry matrix with. In recent years, others have reinvented MCA [7–9], using different notation, highlighting the fact that there has been a good deal of conceptual convergence in this area.

Although some aspects of MCA had been published previous to the original primary publications [10,11], the papers by Kacser, Burns, Heinrich and Rapoport were focused on how metabolites and fluxes in metabolic pathways are affected by changes in enzyme activities and other external factors. The work by Savageau [1], in contrast, emphasized the effect on species concentrations with no mention of fluxes but also uniquely discussed the dynamic stability of dynamical models. The article by Kacser & Burns [2], titled ‘The control of flux’, was particularly influential because it presented its ideas in a clear, readable and mathematically accessible way. It spoke directly to the community of biochemists and molecular biologists, who only had a passing familiarity with differential calculus and, at that time, were largely non-quantitative.

Even though the Kacser & Burns article was clearly written, it was ignored for many years (as was the work by Heinrich & Rapoport [4] and Savageau [1]). Only in the early 1980s did the work begin to be noticed particularly by individuals such as Groen, Wanders, Westerhoff and coworkers at the Amsterdam school [12]. At this point, the paper’s impact rose rapidly and remained highly cited for the next 15 years and continues to be cited today at a rate of about 20 citations per year. To date (December 2023), according to Google Scholar, the article has been cited 2622 times. Similar numbers can be found for the work by Heinrich & Rapoport [4], Savageau [1] and their coworkers. There have of course been criticisms, always unfounded, due to misunderstanding the theory; either because it could not handle large changes, had no predictive value or simply could not be true [13]. Many of the conclusions made by MCA went against orthodoxy and even today there is resistance. However resistance is often for the wrong reason. MCA certainly does make claims but these are based on a deductive approach using fundamental and largely accepted statements about the nature of enzymes and chemical kinetics. Rather than dismiss the claims of MCA, the scientific approach would be to refute the claims by experimentation. As far as I am aware, not a single result in MCA has been refuted by experimental observation, quite the contrary. This is not to say that MCA is therefore true, that would be a ridiculous statement to make. However, until a better explanation and predictor of the behaviour of cellular pathways emerges, MCA is currently the best we have.

One idea that has caused the most consternation, even today, is the ingrained idea of the rate-limiting step. Interestingly, prior to the 1960s, there was an active literature that spoke against the idea, of what was then called, the ‘master reaction’. The paper by Burton [14] is a good example but there are others. Burton’s paper alone, published in 1936, should convince anyone that the idea of a rate-limiting step makes no logical sense. Somehow in the 1960s, and perhaps earlier, experimentalists took the opposite view and saw metabolism in very simple terms where one only had to identify the single step that controlled the pathway in order to understand it.

Interestingly, the revival in the argument against the idea of the rate limiting step arose entirely from experimental observations of the arginine pathway in *Neurospora* at Edinburgh in the Waddington school; but by geneticists, not biochemists. Fig. 5 in one of the early publications by Kacser & Burns [11], shows how various doses of enzymes in the arginine pathway had little or no effect on growth. As an anecdote, Kacser once told me, while I worked with him in Edinburgh, that what inspired his interest was the observation that *Neurospora* could lose 95% of a given enzyme and appear virtually unchanged phenotypically. This work was presented in a PhD thesis by Tateson in 1972 [15] under the guidance of Waddington, Falconer and Kacser and is available online at the Edinburgh thesis archive.

My own experience of MCA was not based on experimental science but on my first attempts to run computer simulations. I thought at the time, that if I could run a computer simulation of say glycolysis, I could understand how it worked. Unfortunately the computer outputted reams of numbers and graphs, which I had no idea how to interpret. Searching the university library, I stumbled upon the work by Kacser and Burns and realized this was the language I needed to interpret what was happening for my simulation. For me personally, this is what was important. MCA helps one understand why a given biochemical pathway has certain phenotype properties based on the properties of the component parts. In a small sense, it bridged genotype to phenotype.

Today there are thousands of papers published on MCA and a growing number of textbooks. There are even pages on Wikipedia that describe MCA.

In relation to other fields, work by Ingalls [16] in 2004 showed that MCA and engineering control theory [17] were one and the same thing. Unlike electrical and mechanical systems, which are the mainstay of engineering control theory, biochemical networks have unique properties such as stoichiometry and mass conservation, which warrant special treatment not found in engineering control theory. This difference is partly manifest in the various summation and connectivity theorems that emerge from the analysis, which give additional insight into the properties of biochemical pathways. There are also definite connections with both the pioneering works of Clarke [18] and Feinberg [19] in the area of chemical network theory but which have yet to be made clear.

To commemorate the 50 years since the publication of the work by Kacser, Heinrich and Savageau, we have received two papers that continue the development of MCA and a historical note from Tom Rapoport [20]. The first paper is by Liebermeister & Noor [21], who describe the use of MCA to discover optimally principles in the distribution of protein across metabolic networks. A second paper by Kochen *et al.* [22] describes how MCA, can provide interesting and useful insights into the dynamic properties of protein signalling pathways. Finally, Tom Rapoport [20] gives a historical perspective on how field developed in Berlin. It is worth mentioning that a special theme is also being published in *Biosystems* and includes additional papers of interest.

## Data Availability

This article has no additional data.
